# 

**Published:** 1890-11

**Authors:** 


CONTENTS:

PAGE

ORIGINAL ARTICLES:

The Wormian and ——? ——Bones. By T. D. Hinebauch . . . . . . . . 597

National and International Meat Inspection. By Olfof Schwartzkoff . . 599

Age of the Horse, Ox, Dog, and other Domesticated Animals. By R. S. Huidekoper, M.D., Veterinarian . . . . . . . . . . . . . . . . . 608

SOCIETY PROCEEDINGS:

Twenty-seventh Annual Meeting of the United States Veterinary Medical Association . . . . . . . . . . . . . . . . . . . . . . . . 613

VETERINARY COLLEGE NOTES . . . . . . . . . . . . . . . . . . . . . . 675

ANNOUNCEMENTS . . . . . . . . . . . . . . . . . . . . . . . . . . . . 676

REVIEW DEPARTMENT . . . . . . . . . . . . . . . . . . . . . . . . . . 677

BOOKS AND PAMPHLETS RECEIVED . . . . . . . . . . . . . . . . . . . . 680

